# Pneumococcal Vaccination in Immunocompromised Hosts: An Update

**DOI:** 10.3390/vaccines9060536

**Published:** 2021-05-21

**Authors:** Claire Froneman, Peter Kelleher, Ricardo J. José

**Affiliations:** 1 Department of Respiratory Medicine, Royal Brompton Hospital, London SW3 6NP, UK; cfroneman@nhs.net (C.F.); P.kelleher@rbht.nhs.uk (P.K.); 2 Department of Infectious Disease, Imperial College London, South Kensington Campus, London SW7 2AZ, UK; 3 Centre for Inflammation and Tissue Repair, UCL, London WC1E 6BT, UK

**Keywords:** immunocompromised, respiratory infection, pneumonia, streptococcus pneumoniae, pneumococcus, vaccination, PCV13, PPV23

## Abstract

Infections with the pathogen, *Streptococcus pneumoniae*, are a common cause of morbidity and mortality worldwide. It particularly affects those at the extremes of age and immunocompromised individuals. Preventing pneumococcal disease is paramount in at risk individuals, and pneumococcal vaccination should be offered. Here, we discuss the role of pneumococcal vaccination in specific groups of immunocompromised hosts.

## 1. Introduction

Pneumococcal disease describes infections caused by the encapsulated Gram- positive bacterium *Streptococcus pneumoniae* [1]. The disease spectrum includes non- invasive disease (e.g., sinusitis, otitis media, and pneumonia) as well as the more serious invasive infection (e.g., meningitis, empyema and bacteraemia) [2].

Pneumococcal disease is a common cause of morbidity and mortality worldwide. The World Health Organisation (WHO) has reported a total of 1.6 million deaths globally caused by *Streptococcus pneumoniae* each year [3]. It has a significant impact in lower-income countries, in populations at the extremes of age and those that are immunocompromised [4]. Even in developed countries, pneumococcal disease remains a significant problem, accounting for more than 250,000 hospital admissions every year, and is associated with severe clinical and economic impacts [5]. Annually, Public Health England reports approximately 5000 cases of invasive pneumococcal disease (IPD) in the United Kingdom.

Currently, there are two common types of pneumococcal vaccines. These vaccines use either a polysaccharide capsular antigen alone (PPV) or conjugated to a carrier protein (PCV) [6]. The current PPV (Pneumovax23) consists of pneumococcal polysaccharide antigens covering 23 serotypes and stimulates a T-cell independent antibody response resulting in no immunological memory [7]. PCV13 (Prevenar) covers against the serotypes in PCV7 (4, 6B, 9V, 14, 18C, 19F, and 23F) and serotypes 1, 3, 5, 6A, 7F and 19A. Conjugated vaccines use modified non-toxic mutant of the Diphtheria toxin as the carrier protein. PCV10 covers against serotypes 1, 4, 5, 6B, 7F, 9V, 14, 18C, 19F, and 23F and uses *Haemophilus influenzae* Protein D, tetanus toxoid, or diphtheria toxoid as the carrier proteins depending on the capsular antigen. Importantly, PCV stimulates a T-cell-dependent response [7,8].

Pneumococcal vaccination is currently indicated in adults with risk factors for severe complications following infection. This group includes a large cohort of people ranging from the very young (0–2 years) to those older than 65 years, those with chronic organ damage (e.g., heart failure and chronic respiratory disease) and immunocompromised hosts. The immunocompromised host includes individuals with primary and secondary immunodeficiencies [9]. The current recommendation in the UK is for the PCV13 to be offered to children as part of the childhood immunisation schedule at the age of 12 weeks and one year. It is also recommended for immunisation of individuals with asplenia, splenic dysfunction, complement disorders, and those severely immunocompromised (e.g., acute/chronic leukaemia, multiple myeloma, stem cell transplantation) and with genetic disorders (e.g., IRAK-4 deficiency) [10]. Furthermore, the PPV23 is offered to those aged 65 years and older. In the US, PCV13 is recommended to all children under the age of 2 years, and either the PCV13 or PPV23 can be considered for those over the age of 2 years with co-morbidities. Since the incidence of PCV13-related disease has declined significantly in adults over 65 years, use of the PCV13 vaccine is unlikely to have a significant impact on the pneumococcal disease in this age group. Hence, the Advisory Committee on Immunization Practices has reversed their recommendation to use PCV13 in all adults over 65 years and now suggests a single dose of PPV23 if there is not a history of being immunocompromised, CSF leak or cochlear implant. If PCV13 is considered, this should be through “shared decision making” and the recommended gap between PCV13 and PPV23 is at least 12 months [11]. For those who are immunocompromised, have a CSF leak or cochlear implant, PCV13 is recommended, with PPV23 given 2 months later.

The sequence of vaccination with PCV13 initially followed by PPV23 has been shown to provide a better response to the pneumococcal serotypes included in both vaccines [12]. A systematic review of 30 publications including 2406 subjects identified the PCV13 vaccine to be safe and largely well tolerated [7]. Specifically, it concluded that concerns of immunogenicity and safety were not supported and should not form a barrier to vaccinating immunocompromised hosts. In the UK, it is recommended that severely immunocompromised people who have already had the PPV23 should wait at least 6 months before being given the PCV13 [9]. ACIP differ in their opinion and recommend 2 doses of PPV23 after PCV13 in the immunocompromised patient group: the first dose 8 weeks after PCV13 and then, the second dose 5 years after the first PPV23 vaccine if the individual is less than 65 years old.

However, an immunological hyporesponsiveness phenomenon has been described following secondary vaccination, particularly with PPV23 [13,14]. There are several mechanisms which have been described to account for this phenomenon and interestingly, this hyporesponsiveness is not exclusively a vaccine-induced phenomenon. Vaccine hyporesponsiveness refers to the inability of individuals to augment immune responses after booster vaccinations or colonisation compared with primary vaccination in the absence of colonisation [15]. This is well recognised following repeated meningococcal polysaccharide vaccination; however, the impact of age, serotype, antigen dose and trial design has resulted in contradictory results for repeat pneumococcal polysaccharide vaccine responses [15]. Vaccine hyporesponsiveness induced by the PPV23 is not sustained in young children and appears to have limited clinical consequences for subjects at high risk of pneumococcal disease [16]. There is no long-term evidence of hyporesponsiveness after use of PCV in children previously immunised with PPV. Vaccination with PCV does not generally exhibit vaccine hyporesponsiveness; however, failure to boost serotype 3 is a notable feature of PCV and may account for the lack of decline in serotype 3-related disease following the introduction of PCV13 into the childhood vaccine schedule [17]. 

Whilst there are benefits of pneumococcal vaccination, particularly at reducing risk of invasive disease, rates of vaccination generally remain suboptimal [5]. Several factors have been identified as potential causes for this, including the uncertainty of responsibility of vaccination between GPs and medical specialists, the uncertainty of optimal vaccination time and concern of deterioration of high-risk conditions due to vaccination. In particular, a cohort study carried out in Germany concluded that vaccination rates remained very low within the 3-year period after the development of a new, high-risk condition specific for pneumococcal disease and that vaccination after this event occurred too late [5].

The objective of this review is to provide an update of pneumococcal vaccination in specific immunocompromised hosts. 

## 2. Primary Immunodeficiency

As of 2019, there are 430 inborn errors of immunity resulting in primary immunodeficiencies [18]. These conditions increase susceptibility to not only infectious diseases but also allergies, malignancies, autoimmune and autoinflammatory conditions [18]. Whilst major antibody deficiency syndromes are managed with immunoglobulin replacement therapy, which contains circulating antibodies to pneumococcal serotypes in the population, and severe combined immunodeficiency treated with stem cell transplantation, a role for pneumococcal vaccination is indicated for disorders of innate immunity associated with increased risk or complications of pneumococcal infection [19]. Furthermore, vaccination with the polysaccharide pneumococcal vaccine is used for diagnostic purposes. However, the intrinsic nature of the immunodeficiency means that interpretation of an antibody response and, therefore, overall immunity may be complex [20]. 

The classification of a successful immunisation relies on functioning humoral immunity with quantitative antibody response. When a poor antibody response is noted, current practice is to provide a further booster vaccination to generate a qualitative antibody response. In this way, it is also possible to provide a quantitative measure of immunity within different populations for subsequent vaccination standardisation within patient populations [20]. 

Within the primary immunodeficiency cohort, it is worth noting that different antibody levels are required to prevent specific infections and it is important to note that the correlates of protection for pneumococcal disease are not well defined. In particular, pneumococcal antibody levels required to prevent sinusitis, otitis, bronchitis and pneumonia have been noted to be greater than those required to prevent invasive pneumococcal disease [20]. 

Individuals with primary immunodeficiency are at risk of pneumococcal disease due to their genetic immunity deficits, and the current UK vaccine recommendation is for these people to be given a single dose of the PCV13 followed by PPSV23 after a period of at least 2 months [9]. As pneumococcal vaccinations are not live vaccines, they are considered safe to administer in patients with immunodeficiencies and certainly, are well tolerated within this cohort [20]. 

## 3. Secondary Immunodeficiency

Secondary immunodeficiencies occur when the immune system is impaired by external variables such as infectious disease, malignancy, immunosuppressive therapy, and severe environmental conditions such as malnutrition [21]. As with primary immunodeficiencies, vaccination within the secondary immunodeficiency cohort may result in little or no protection due to the overall impairment of the immune system, and needs to be assessed on an individual basis. 

Pneumococcal vaccination is indicated in all patients with secondary immunodeficiency. The current UK recommendation remains for the PPV23 because of its extended serotype coverage and the calculated low cost-effectiveness of PCV13. However, as mentioned previously, the immune response generated is T cell independent, inducing an IgM-dominated antibody response without immunological memory. This results in waning of immunity after 2–4 years and in high-risk individuals between the age of 19 and 64 years, the CDC recommends a second dose of PPV23 5 years after the first dose of PPV23 [10,11,13,22]. Those 65 years and older who received PPV23 should receive a final dose of PPV23 at age 65 or older at least 5 years since the last vaccine [10,11]. The PCV13 is highly immunogenic and thereby, provides higher antibody titres and greater immunological memory. The conjugation of the polysaccharide antigen to a carrier protein induces a T-cell-dependent immune response and is recommended in the UK for people with secondary immunodeficiency if the measured antibody response post-PPV23 is inadequate. This increased immunogenicity is the basis for sequential administration of PCV13 following the PPV23 [22]. The CDC recommends the PCV13 for individuals 19 years and older who are immunocompromised [11].

### 3.1. Human Immunodeficiency Virus Infection

The risk of invasive pneumococcal disease is up to 100 times higher in the HIV population not on antiretrovirals (ART) than in an age matched uninfected population [23]. There is an additional increase in recurrent invasive infections post treatment of pneumococcal disease [23]. This has reduced significantly following the advent of combined ART [24], however this incidence remains elevated in HIV populations when compared to a general population [25]. Risk factors for invasive pneumococcal disease within the HIV population include ethnicity, CD 4 count (200–499 cells/mL), intravenous drug and excess alcohol use, a smoking history and chronic liver disease [26,27]. This has been shown to occur even in patients who have adequate viral suppression and high CD 4 counts, which is influenced by the nadir count before starting ART and failure to restore IgM memory B cell subsets in those who start ART when CD4 counts less than 350 cells/UI [25].

The use of a pneumococcal vaccine for people with HIV has therefore been recommended since early on in the HIV pandemic [28]. However, the overall efficacy of the vaccine has been debated for some time. The PPV23 is known to have reduced efficacy in HIV infected adults compared to PCV and PPV23 is therefore not recommended for HIV patients in some areas of the world [29].

A study of 977 adults who had survived a confirmed invasive pneumococcal event and who subsequently received two doses of the PCV13 were further investigated for PCV13 efficacy as well as recurrence of invasive pneumococcal disease [23]. The study found that use of two doses of the PCV13 prevented 74% of recurrent invasive pneumococcal disease events. The vaccine was noted to be most efficacious during the first 12 months post vaccination and prevented disease when CD4 counts were below 200 cells/uL. 

Although evidence is lacking, the current UK recommendation is for sequential vaccination with PCV13 followed by PPV23 to induce and maintain protective immunity [30]. There is growing evidence to delay PCV13 vaccination until after recovery of CD4 T cell count (>200 cells/mm^3^) due to the immunology of T- cell dependent versus T- cell independent vaccines [24]. As treatment with cART improves longevity in the HIV population, further studies are needed to examine the durability of the immune response against pneumococcal disease. This is increasingly important as the severity of pneumococcal disease increases with increasing age [25]. The current recommendation by the infectious disease society of America (IDSA) is for a booster dose 5 years after initial vaccination and although it may be local practice, it is not currently recommended to check antibody titres [31].

### 3.2. Autoimmune/Rheumatologic Disease

People with autoimmune or rheumatologic disease may be immunocompromised with a higher risk for infections. Indeed, cohorts of these individuals are noted to be as much as 7 times as likely to be hospitalised with an infection than the general population [32,33]. The use of immunosuppressive drugs is, however, an important confounding factor. The effect of immunosuppressive medication is discussed below.

Most studies on the vaccination of patients with rheumatological disease have shown a largely normal response to vaccination [34]. However, there is evidence that certain disease-modifying agents can impair the vaccine response, requiring further monitoring of antibody levels including methotrexate and anti-TNF alpha therapies [35,36]. Despite the substantial evidence of safety of vaccination against pneumococcal disease, a study of rheumatology outpatients found that vaccination rates were suboptimal, despite adequate patient awareness [37]. A further study of 1033 patients identified several interventions to improve immunisation rates, including an immunisation algorithm and an immunisation clinic reminder which improved rates at least three-fold over a period of 53 weeks [38]. Due to the immunosuppression associated with autoimmune and rheumatological disease, the current vaccine recommendation is for the PCV13 followed by the PPSV23 2 months later [9]. It is recommended that ideally, vaccination should be given at least 2 weeks prior to initiation of immunosuppressive medication.

### 3.3. Immunosuppressant Medication

Studies have shown that people treated with immunosuppressive agents show a reduced response to vaccinations compared to those not receiving these agents [39]. As immune response can be difficult to quantify, a surrogate marker of antibody titre following vaccination is often used to gauge host response.

However, poor vaccine immune responses are not true for all of the immunosuppressive medications. In particular, upon comparing antibody titres in patients being treated with TNF alpha blockers and those with methotrexate, methotrexate was noted to result in a lower antibody response [36]. These individuals received the PCV7 with IgG antibodies specific to *S. pneumoniae* capsular polysaccharides measured pre-vaccination and at 4–6 weeks following vaccination. The group that received TNF alpha blockers had reduced antibody responses numerically but not statistically when compared to non-treated controls. Additionally, in patients receiving TNF blockers and methotrexate, antibody titres responded similarly to those on methotrexate alone. The study concluded that patients who are to be initiated on methotrexate therapy should have vaccinations given prior to initiation [36].

Other studies on subjects with rheumatic disease have highlighted that response to vaccination is not affected by immunosuppressive therapy [34]. This seems to contrast patients with underlying inflammatory bowel diseases (IBD) who are noted to have a reduced response to vaccinations [34]. This has been reported as resulting from a higher dose of immunosuppressive medication that patients with IBD receive [34]. In contrast, a more recent study of pneumococcal vaccination in people with autoinflammatory diseases including individuals with inflammatory bowel disease on biological therapy demonstrated that vaccination with PCV13 followed by PPV23 resulted in approximately 50% of individuals achieving functional antibodies to the tested serotypes (1, 3, 7F, 14, 19A, and 19F) [40]. This study highlighted the importance of ensuring that immunocompromised individuals receive a correct protocol of vaccination with both PCV13 and PPV23 and that measuring functional antibodies may provide a better measure of immune response to vaccination than just measuring IgG levels. Importantly, this study also demonstrated that immune responses to vaccination in this cohort were likely to be more affected by adalimumab than etanercept, that concomitant use of methotrexate did not impair the functional antibody response compared to those not on methotrexate, and that even low-dose prednisolone (<20 mg per day) had an impact on the functional antibody response [40].

Studies examining the response to pneumococcal vaccination in patients receiving rituximab have shown a significant impairment in antibody response for at least 6 months following treatment with rituximab and in clinical practice, the observation is that the impairment is sustained longer [41,42]. This does not appear to occur with all vaccinations and further studies of antibody response to tetanus toxoid and the *Haemophilus influenzae* vaccination have been noted to be preserved when compared to pneumococcal vaccination response [43]. As a result of the impairment, patients may benefit from immunisation prior to rituximab infusion or between rituximab courses with at least 6 months duration from the last dose [42].

Several studies have been performed to determine the time at which antibody levels reduce in patients on immunosuppressive therapy. Previous studies have shown variation as early as 6 months to 5 years [44]. A 2015 study of subjects with pulmonary diseases receiving immunosuppressive therapy and steroids found that elevated antibody levels persist for 3 years after vaccination [45]. The study concluded that patients on immunosuppressive medication should be revaccinated at a period of 3 years. 

### 3.4. Haematological Malignancies

With the onset of a haematological malignancy, the risk of infection increases due to various deficiencies of humoral- and cell-mediated immunity. This is a result of both the malignant process as well as the administered disease therapy [46]. The incidence of IPD in patients with haematological malignancy ranges between 13 and 50 times higher than that of the background population [47]. Patients with haematological malignancy have been noted to account for approximately 10% of all IPD events in adults [47].

A recent cohort study of 13,332 episodes of first IPD found that the rate of IPD in patients with any malignancy was highest during the first 2 years following diagnosis [48]. For patients with non-haematological malignancies, the incidence of IPD rapidly reduced after the first year of diagnosis. For patients with Chronic Lymphocytic leukaemia, Non-Hodgkin lymphoma and Multiple Myeloma, the incidence of IPD remained elevated or even increased over time.

Following the introduction of the PCV13, a reduction of 9% was noted in the annual incidence of IPD in patients with haematological malignancy [48]. This compared to a reduction of 3.5% in the adult population without haematological malignancy. This highlights the critical importance of vaccination within this highly susceptible cohort of patients. 

There is evidence that vaccination prior to transplant influences the antibody response post-transplant [49]. In a study of antibody response to pneumococcal vaccination following autologous haematopoietic stem cell transplant, patients who received the PCV7 before stem cell collection had significantly higher antibody concentrations at 3 and 6 months when compared to those patients who received the vaccine prior to transplantation [50].

The current recommendation for adults newly diagnosed with a haematological malignancy is to receive PCV13. In the US, the recommendation is that PPV23 should be administered eight weeks after the PCV13 in adults and children >2 years old [31]. In the UK, the recommendation is for the administration of PCV13 in adults and children 5 years or over followed by PPV23 8 weeks later. For individuals with Leukaemia, the recommendation is to wait 6 months after chemotherapy or 9 to 12 months after stem cell transplantation before administering pneumococcal vaccination. Vaccination uptake in cohorts with haematological malignancy remains suboptimal, despite multiple opportunities within the patient journey [51]. 

### 3.5. Solid Organ Transplantation

The incidence of IPD is noted to be increased in solid organ transplant recipients. A prospective population study demonstrated the incidence of IPD to be 146 per 100,000 transplanted patients per year [51]. This compared to an annual incidence rate of 11.5 per 100,000 in the non-transplanted group. Within the transplant group, the incidence was noted to be the greatest in the liver transplant recipients (354 per 100,000 persons annually), possibly because of the regimen of immunosuppressive medication as well lack of prior vaccination or suboptimal vaccination response [51]. The overall rate of vaccination within the transplant group is known to be suboptimal [48]. An inhibiting factor to uptake of vaccination within the transplant group may be a result of the uncertainty of the effectiveness of the PPV23 as well as concerns of vaccine safety. Despite this, there is evidence that patients with solid organ transplantation derive benefit from the administration of vaccines [52]. If possible, vaccination should occur prior to transplantation in order for the patient to derive maximum protection from the vaccination [53]. Additionally, further studies have shown that vaccination against pneumococcal disease does not increase the risk of alloresponse such as organ rejection or the development of specific antibodies [52].

### 3.6. Immunosenescence and Ageing

In adults, *S. pneumoniae* mainly affects those >65 years of age, and this age-related increased risk is independent of comorbidities and health status [54,55,56]. CAP in the elderly is associated with increased morbidity, use of healthcare resources (estimated at GBP 1 billion/year in the UK), functional decline and high mortality [55,57]. Ageing results in impaired functioning of the immune system and is particularly evident after 75 years of age. There are many factors that impact the overall functioning of the immune system in older age. These include defects in haematopoietic bone marrow to defects in peripheral lymphocyte migration, maturation and function, and age-related thymus atrophy, overall resulting in an impairment in both the innate and adaptive immune response [58]. As is well known, the ageing population has an increased susceptibility to infection for a plethora of reasons, resulting in increased morbidity and mortality [59]. The resultant impairment in the immune system is crucial for vaccination, for which the overall success depends upon a functioning immune system [60].

In contrast to the effect of pneumococcal vaccination in childhood, vaccination in the elderly population provides only a mitigated response to pneumococcal infection rather than full protective immunity [61]. The ageing response to vaccination provides a general conundrum. A large proportion of research in the field of vaccination is carried out on younger populations. Not only this but as immunosenescence occurs, several other factors impact the general health of the host. This includes an increased incidence of autoimmunity which, as discussed earlier, has a significant impact on vaccination response. The strategy for an improvement in the response to vaccination is, therefore, multifactorial and includes improving memory immunity earlier on in adult life [60]. The global and economic implications into successful vaccination of the ageing population cannot be understated and should be prioritised in general research. For now, the UK continues to recommend the PPV23 for adults over the age of 65 years to reduce IPD, knowing that it has little impact on non-invasive disease such as pneumonia and exacerbations of COPD, mainly due to the high cost of PCV, making it not cost-effective. In the US, however, PCV13 is recommended with consideration of PPV23 8 weeks or 1 year later for those 65 years and older, with or without an immunocompromising condition, respectively.

### 3.7. Chronic Organ Disease

#### 3.7.1. Kidney

Patients with renal disease have increased susceptibility to infection for a multitude of reasons including immunosuppressive medications and invasive procedures (vascular access catheters) [62]. Innately, the uraemic state of renal disease also results in a reduced host defence, affecting neutrophil function, antigen processing and antibody formation [62]. Additionally, patients with end stage kidney disease have a reduced response to vaccines with lower antibody titres following vaccination when compared to non-dialysed patients [62]. This seems to be independent of the dialysis type and correlates more directly with the degree of renal failure [63].

It is counterintuitive, therefore, that vaccination rates within the renal disease population are reduced [62,64], but there is conflicting evidence regarding the use of PPV23 in renal patients. A retrospective cohort study found that there was no difference in the rate of hospitalisation due to pneumonia between the vaccinated and non-vaccinated patient cohorts [65]. However, PPV23 vaccination significantly improved prognosis for patients on dialysis. This was concluded to be secondary to the overall reduction in cardiovascular events, which are known to occur in conjunction with pneumococcal pneumonia [65]. Importantly, a significant association has been found between pneumococcal immunoglobulins resulting from PPV23 and anti-oxidised LDL (oxLDL) antibody titres, suggesting that pneumococcal vaccination may induce anti-oxidised low-density lipoprotein which is cardioprotective [66]. This is not limited to renal patients and has been found to occur in the cardiac cohort of patients [66]. In the UK, the recommendation is for administration of PPV23, which should be repeated every 5 years [9], whilst in the US, the CDC recommendation is for one dose of PCV13 followed by PPV23 8 weeks later and another dose of PPV23 after 5 years. 

#### 3.7.2. Liver

The role of immunisations in people with chronic liver disease cannot be understated. Despite this, robust studies of pneumococcal disease in chronic liver disease remain scarce. The majority of these studies have been restricted to patients with chronic hepatitis B and C, alcoholic liver disease, compensated and decompensated cirrhosis, and liver transplant recipients.

Similarly to chronic renal failure, patients with chronic liver disease show a reduced immune response to vaccinations and an increased susceptibility to pathogens [67,68]. Common reasons for reduced immunity following vaccination include both immunosuppressive medication and advanced liver disease [68].

Few studies have been undertaken to determine the antibody response to pneumococcal vaccination in individuals with chronic liver disease. A study of 45 adults with end stage liver disease were vaccinated with PPV23, with subsequent evaluation of an antibody response [69]. Antibody levels were measured at 1 and 6 months after vaccination and patients who received transplants were evaluated at the time of transplantation and 3 months following transplantation. In all of the patients, antibody levels declined more rapidly than control subjects during the first 6 months following transplantation. The subjects who received liver transplants also showed reduced antibody levels at 3 months following transplantation [69]. Further research is required into vaccine effectiveness for patients with chronic liver disease.

PPV23 is recommended for use in adults with chronic liver disease as it specifically confers protection against infections such as peritonitis caused by *Streptococcus pneumoniae*, the third most common cause of spontaneous bacterial peritonitis [70].

#### 3.7.3. Heart

The PPV23 vaccine has been shown to have a cardiac protective effect. This is a result of a reduction in atherosclerosis; however, the exact mechanism for this reduction is not well understood at present. It is thought that vaccination with the polysaccharide vaccine leads to the production of antibodies that cross-react with oxLDL, a well-known component of atherosclerotic plaques. These antibodies exert a cardioprotective effect in three potential ways: (1) The bound antibodies may prevent the formation of foam cells by blocking macrophage uptake of oxLDL. (2) Alternately, the antibodies result in the clearance of the atherosclerotic plaques by binding on apoptotic cells within the plaque. (3) Finally, it is postulated that the antibodies may neutralise the proinflammatory cascade, which is induced by oxLDL [71]. Furthermore, it is known that during and following an episode of pneumococcal pneumonia, individuals have an increased risk of cardiovascular complications including arrythmia, myocardial ischaemia and heart failure [72] in addition to increased cardiovascular-related mortality for a year following discharge with pneumonia [73].

*Streptococcus pneumoniae* can directly result in cardiac events through several pathogenic mechanisms including the translocation of *S. pneumoniae* into the myocardium. This may account for the disrupted electrophysiology and increased arrhythmic events [73]. As the lesions spread into adjoining cardiomyocytes, this may further reduce the overall capacity of the myocardium to contract. It also hypothesised that *S. pneumoniae* produces a cholesterol-dependent metabolite, cytolysin, which disrupts contractility through unregulated calcium entry into cardiomyocytes. Specifically, pneumolysin, a member of the cholesterol-dependent cytolysins, is recognised to have a key involvement in the pathogenesis of myocardial injury [74,75]. Amongst several mechanisms, this direct cardiotoxic activity occurs through myocardial invasion and necroptosis of cardiac macrophages, which results in further microlesions [76]. Vaccination against pneumococcal disease has been shown to provide a significant reduction in cardiac events [77].

Therefore, prevention of pneumococcal pneumonia in an already at-risk population is of utmost importance and in the UK, PPV23 is recommended to all adults with chronic heart disease. 

### 3.8. Asplenia/Sickle Cell Disease

The spleen plays a critical role in the removal of bacteria from the blood stream. As blood circulates through the pulp of the spleen, splenic macrophages remove opsonised bacteria. Encapsulated bacteria are poorly opsonised as the polysaccharide capsule hinders binding of the complement and prevents the complement from interacting with the macrophage. Additionally, the spleen is responsible for producing IgM memory B cells, which are important in subsequent recognition of encapsulated bacteria [78].

Asplenia refers to complete loss of function of the spleen, which may be anatomical or functional (i.e., surgical splenectomy or loss of function due to medical conditions such as sickle cell anaemia) [78]. Overwhelming post-splenectomy infection (OPSI) refers to a syndrome of fulminant sepsis occurring within 24–48 h of an initial presentation of viral-type symptoms [79]. The encapsulated organisms *S. pneumoniae*, *N. meningitides* and *H. influenza* are typically responsible in this cohort of patients [78]. 

As of 2021, UK NICE guidelines recommend a single dose of PCV13 followed by PPV23 after a period of 2 months for patients with asplenia or splenic dysfunction [9]. Similarly to chronic kidney disease, patients with asplenia should be revaccinated with PPV23 every 5 years. For patients who will be undergoing a splenectomy, vaccination should be offered 4–6 weeks before hand. 

## 4. Pneumococcal Vaccination for the Diagnosis of Immunodeficiency

From a young age, humans are exposed to pneumococcal serotypes, with colonisation of the nasopharyngeal tract seen in 40% or more of children [80]. Therefore, even outside the context of vaccination, immunocompetent individuals should have detectable levels of total pneumococcal antibodies. If pneumococcal antibody levels are low or specific serotype levels are below the currently recommended protective level for IPD, then individuals should be immunised to assess their vaccine response and to afford these individuals protective antibody levels if they mount a good response. Currently, the threshold for protective serotype-specific antibody level is 0.35 μg/mL for the PCV13 and it is expected that post-vaccination antibody levels should be >0.35 ug/mL for at least 7 out of the 13 serotypes found in PCV13 [81]. It should be stressed that the threshold value is a composite value for all vaccine-related serotypes; however, in practice, thresholds are likely to vary with serotype and the concentration for protection against invasive and non-invasive disease and colonisation will also differ [82]. The protective serotype-specific antibody level following PPV23 is considered to be 1.3 ug/mL [81]. 

The most widely use technique utilised to measure anti-pneumococcal antibodies is the enzyme-linked immunosorbent assay (ELISA) [18]. This provides a quantitative measure of this response and provides evidence as to whether the patient can mount an antibody response to the polysaccharide vaccine. However, a functional measure may prove to be more clinically useful. To this end, in vitro opsonophagocytosis assays (OPAs) were developed to provide a functional correlation of immune protection [83] and are now encouraged as the primary analysis of vaccine efficacy in the elderly population, who may have waning antibody levels. 

## 5. Future Strategies for Pneumococcal Immunisation of Immunocompromised Hosts

Current PCVs do not provide protection against non-vaccine serotypes and can select for un-encapsulated *S. pneumoniae* strains. A number of extended serotype pneumococcal protein conjugates are under investigation in Phase 3 trials, including PCV15 (V114) and the 20 valent PCV (20nPVC) [84]. Such vaccines however are likely to overcome the problem of replacement serotype disease or the emergence of unencapsulated pneumococcal variants. Ultimately, a universal pneumococcal vaccine independent of serotype is required to address these issues. Promising universal vaccine candidates include *S. pneumoniae* killed whole cell vaccine (WCV), or pneumococcal antigens derived from highly conserved proteins or virulence factors (pneumococcal surface protein A, pneumococcal histidine triad protein D, pneumococcal surface adhesion A, and detoxified pneumolysin variants). There is considerable interest in the application of nanoparticles and bacterium-like particles as vehicles to promote mucosal immune responses and to enhance the immunogenicity of candidate pneumococcal protein vaccines. In addition, strategies to improve the performance of current pneumococcal vaccines in the elderly include increased vaccine dose [85] and the deployment of modern adjuvants [86]. For example, adjuvant system AS02V enhances humoral and cellular immune responses to the pneumococcal protein PhtD vaccine in healthy young and older adults. Based on vaccines using conserved pneumococcal peptide sequences or lipoprotein antigens, the immunogenicity of peptide sequences will most likely be increased by novel antigen delivery systems and vaccine adjuvants [87].

## Data Availability

No required.
